# Assessing Feed-Forward Backpropagation Artificial Neural Networks for Strain-Rate-Sensitive Mechanical Modeling

**DOI:** 10.3390/ma17020317

**Published:** 2024-01-08

**Authors:** Víctor Tuninetti, Diego Forcael, Marian Valenzuela, Alex Martínez, Andrés Ávila, Carlos Medina, Gonzalo Pincheira, Alexis Salas, Angelo Oñate, Laurent Duchêne

**Affiliations:** 1 Department of Mechanical Engineering, Universidad de La Frontera, Temuco 4811230, Chile; d.forcael01@ufromail.cl (D.F.); a.martinez16@ufromail.cl (A.M.); 2 Doctoral Program in Sciences of Natural Resources, Universidad de La Frontera, Temuco 4811230, Chile; m.valenzuela16@ufromail.cl; 3 Centro de Excelencia de Modelación y Computación Científica, Universidad de La Frontera, Temuco 4811322, Chile; andres.avila@ufrontera.cl; 4 Department of Mechanical Engineering, Faculty of Engineering, University of Concepción, Concepción 4070138, Chile; cmedinam@udec.cl (C.M.); alesalas@udec.cl (A.S.); 5 Department of Industrial Technologies, Faculty of Engineering, Universidad of Talca, Curicó 3340000, Chile; gpincheira@utalca.cl; 6 Department of Mechanical Engineering, Faculty of Engineering, Universidad del Bío-Bío, Concepción 4081112, Chile; aonates@udec.cl; 7 Department of Materials Engineering (DIMAT), Faculty of Engineering, Universidad de Concepcion, Concepción 4070138, Chile; 8 Department ArGEnCo-MSM, University of Liège, 4000 Liège, Belgium; l.duchene@uliege.be

**Keywords:** modeling, mechanical behavior, plastic flow, strain rate, artificial neural network

## Abstract

The manufacturing processes and design of metal and alloy products can be performed over a wide range of strain rates and temperatures. To design and optimize these processes using computational mechanics tools, the selection and calibration of the constitutive models is critical. In the case of hazardous and explosive impact loads, it is not always possible to test material properties. For this purpose, this paper assesses the efficiency and the accuracy of different architectures of ANNs for the identification of the Johnson–Cook material model parameters. The implemented computational tool of an ANN-based parameter identification strategy provides adequate results in a range of strain rates required for general manufacturing and product design applications. Four ANN architectures are studied to find the most suitable configuration for a reduced amount of experimental data, particularly for cases where high-impact testing is constrained. The different ANN structures are evaluated based on the model’s predictive capability, revealing that the perceptron-based network of 66 inputs and one hidden layer of 30 neurons provides the highest prediction accuracy of the effective flow stress–strain behavior of Ti64 alloy and three virtual materials.

## 1. Introduction

Manufacturing processes of metal and alloy products operating under explosive and hazardous impact loads can be designed and optimized using computational mechanical tools based on mathematical modeling. The processing variables such as strain, strain rate, and temperature of the various existing manufacturing processes can considerably change, from low to moderate and even to high or extreme values [1,2]. An accurate design requires testing and defining the real behavior of the material being used, as well as calibrating an implemented mathematical model capable of reproducing the mechanical behavior in a computational tool generally based on the finite element method [3,4,5,6,7]. Direct numerical calibration of model parameters with experimental flow curves is commonly performed using the well-known generalized reduced gradient method [8,9], derivate-free algorithms [10], or Levenberg–Marquardt-type approaches [11]. For higher accuracy in predicting the mechanical behavior of materials under different deformation scenarios, and including other mechanical features such as anisotropy [12], strength differential effect [13], kinematic hardening [7], or damage [14], inverse and mixed strategies based on numerical modeling are generally preferred. For instance, Rojas et al. [12] calibrated the elastoplastic model of Ti64 at nanometer-length scales using a hybrid finite element and experimental technique based on nanoindentation. The reported calibration results were highly accurate for the investigated quasi-static and room temperature response.

Despite the fact that direct and inverse approaches are generally chosen for the specific calibration of mechanical models describing the plastic flow behavior of metals and alloys, artificial neural networks (ANNs) are gaining interest in several fields of research and industry as an automatic tool for enhancing recognition, identification, and prediction. Inspired by the homologous behavior of biological neurons, ANNs learn and train themselves rather than being explicitly programmed. In the study of the mechanical behavior of materials, ANNs can be applied to directly predict behavior [15,16,17] or to optimize and assist in finding the parameters of material models [15,18,19]. When an ANN-based identification method is well-trained on various cases, it can be expected to be more robust and versatile than classical direct and inverse techniques, which will always require a skilled human supervision. In Sun et al. [20], a backpropagation learning algorithm with a 3-12-1 ANN configuration was efficiently applied by correlating the processing parameters and flow stress of the Ti-6Al-2Zr-1Mo-1V alloy. In Lee et al. [15], an inverse approach was reported to successfully combine an ANN with finite element (FE) modeling to extract the macroscopic stress–strain values of ordinary Portland cement paste samples from nanoindentation testing. Xie et al. [21] applied the backpropagation artificial neural network combined with the phase field technique for predicting the residual lifetime of mechanical structures. Recently, ANN-based hybrid models have been featured as a promising approach for predicting the behavior of materials in a physical sense. In Kalina et al. [17], the flow stress of isotropic hyperelastic materials is accurately computed from the experimentally trained ANN combined with the classical well-established constitutive Ogden’s model, previously informed from reduced data space and adjusted weights using the thermodynamics consistency approach. 

Several proposed ANN strategies require the definition of specific architecture features and quality training data to obtain accurate prediction results. Indeed, adding numerically generated results from FE or complex experimental data improves predictions, although it is time-consuming and rarely straightforward. In addition to the type of neural network techniques previously discussed, other studies include the memory effect on recurrent neural networks (RNNs) for modeling plastic flow with different deformation path dependence [22]. By contrast, others have shown convolutional neural network techniques with constrained equilibrium conditions. Using training input data such as the surface field of deformation and external force, the constitutive behavior of the material flow stress and deformation was adequately modeled [23].

The objective of this paper is to assess the efficiency and the accuracy of different architectures of ANNs for the identification of material parameters for the Johnson–Cook material model at various strain rates and temperatures.

### 1.1. Dynamic and Static Flow Stress with Thermal Softening of Ti64

**Figure 1:**
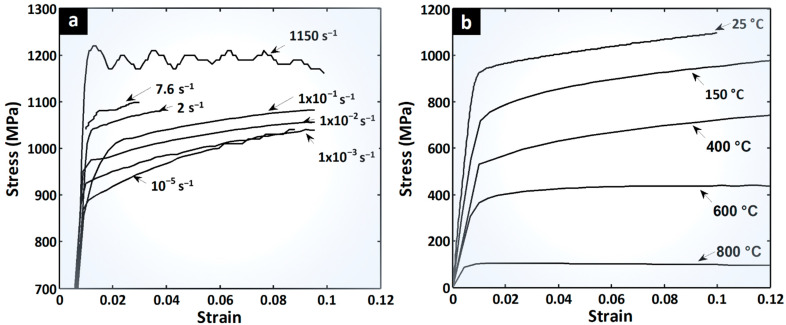
Flow behavior of the Ti64 (**a**) between 10^−5^/s and 10^3^/s at 25 °C and (**b**) thermal softening at 10^−3^/s from 25 °C to 800 °C.

The uniaxial tensile flow stress behavior was previously determined for the same material at different laboratories. For the strain rates testing at 10^−5^, 2, and 7.6 per second, a miniature tensile test sample with a diameter of 3 mm was used by Lecarme [24] (Figure 1a). The static range between 10^−3^ and 10^−1^ s^−1^ and thermal curves (Figure 1b) are from Tuninetti et al. [25] using a cylindrical sample with a diameter of 6 mm. The highest strain rate curve has been obtained from a Kolsky bar tensing facility and high speed cameras at Ghent University and the results are available from Peirs [26]. In the present work, the data provided in Figure 1 are used to test and validate the ANN. 

This work presents the implementation strategy of a computational tool for the parameter identification of mechanical modeling of flow behavior sensitive to strain rate and temperature. The proposed strategy and different ANN configurations are evaluated on three virtually generated materials and a known Ti64 alloy [27]. The range of interest of the Ti64 deformation process parameters investigated here has been extended from previously published work, covering the full range of deformations, temperatures, and strain rates that the material commonly withstands in applications such as machining and forming processes, as well as for product design for safe operation under extreme conditions such as bird strike in jet engines.

The proposed novel ANN-based calibration technique of flow stress applied to Ti64 in the dynamic and quasi-static regimes, including thermal softening, is performed using an original workflow. The testing of four different feed-forward architectures, primarily trained with constitutive flow equations for a wide parameter spectrum, are consequently assessed with an experimental dataset of flow curves. The application of new techniques for calibration or identification of mathematical models of the mechanical behavior of materials remains a relevant subject in mechanical and civil engineering research, as has been demonstrated in previous research [16,17,20,28,29,30,31,32], with particular focus on the simplicity, accuracy, and robustness.

### 1.2. Mechanical Model of Flow Stress Behavior

Typical strain rates in machining operations are in the extreme order of 10^3^ s^−1^ [33], whereas for metal-forming processes such as forging, rolling or extrusion, and superplastic forming, the values range from 10^−4^ to 10^3^ s^−1^ [1]. Lower values of strain rates of about 10^−5^ s^−1^ have been investigated in gum metal titanium alloys for automotive process applications [34]. This work investigates flow behavior for three virtual materials and an aerospace titanium-based alloy (Ti64) between 10^−3^ and 10^3^ s^−1^.

The mathematical expression to describe the mechanical flow behavior of Ti64 is given by the accepted Johnson–Cook (JC) model (Equation (1)).

(1) $$\bar{\sigma }=\left(A+B{\varepsilon_{p}}^{n}\right)\left[1+C\cdot ln\left(\frac{\dot{\varepsilon }}{\dot{\varepsilon_{0}}}\right)\right]\left(1-{T^{*}}^{m}\right)$$

The direct relationship between the flow resistance ($\bar{\sigma })$ of the material with plastic strain $\left(\varepsilon_{p}\right)$ at a reference strain rate $\left(\dot{\varepsilon_{0}}\right)$ and at a reference temperature ${(T}_{ref}$) is described by the parameters *A*, *B*, *n*. The parameter *C* identifies the sensitivity of the material to the strain rate and *m* takes into account the softening or inverse relationship of flow resistance with the material homologous temperature $\left(T^{*}=\left(T-T_{ref}\right)/\left(T_{fusión}-T_{ref}\right)\right)$. T is the temperature of the material being deformed, while $T_{fusión}$ is the melting temperature of the material. In the deformation process, this model assumes that the $\varepsilon_{p},\;\dot{\varepsilon },\;and\;T$ during the deformation process independently affect the strain hardening rate (multiplicative terms in flow stress). During the last years, several authors have used Johnson–Cook model to predict the flow stress behavior of metals [35,36,37,38]. Ning and Liang [36] calibrated the model parameters with an analytical method based on a modified chip formation model and experimental data of force and temperature of one orthogonal machining test. Chen et al. [39] modeled the deformation and damage of aluminum structures due to soil explosions. The accurate validations demonstrate that this is a well-established mathematical expression to describe material behavior sensitive to both temperature and strain rate during the deformation process.

## 2. Model Calibration Strategy Based on Feed-Forward Backpropagation Neural Network

An architecture of artificial neural networks (ANNs) has been developed to enhance the identification strategy of a Johnson–Cook model that characterizes the strain rate behavior of Ti64 under low, moderate, and high deformation rates, as well as different temperatures. The choice of the ANN methodology is rooted in its capacity to learn from input data without requiring explicit programming, making it an ideal tool for the automated estimation of the constants in the Johnson–Cook model. It is noteworthy that this methodology has demonstrated outstanding results in material parameter identification. The entire process, encompassing data generation strategy, training within the developed architecture, and subsequent validation, has been structured in matrix form and implemented using the Python programming language. The database employed has been generated from theoretical curves of a Ti64 alloy [27,40] and virtual materials with different constant values, at several strain rates and temperatures. In total, seven parameters have been addressed in this process (*A*, *B*, *C*, *n*, *m*, $\dot{\varepsilon },\;and\;T$). As a final step, various ANN configurations have been compared by assessing their model prediction capabilities. This analysis determines the configurations that best fit the characteristics of the problem and provide the best performance in estimating the constants within the Johnson–Cook model. The following subsections provide a detailed description of the methodology.

### 2.1. Modeling of Feed-Forward Neural Networks

Inspired by the behavior of biological neurons, ANNs can learn by themselves without being explicitly programmed. Inputs (*x_j_*) multiplied by weights (*w_j_*) adjust the output values, while middle layer neurons process this information. The output is compared to expected values, and the error is used to modify the initial weights through backpropagation. The perceptron [41] is the most basic neural network. For complex decisions, neural networks with multiple layers composed of multiple perceptrons are required (Equation (2)):

(2) $$Output=\left\{\begin{array}{c} 0\;if\;w\cdot x+b\;\leq 0\\ 1\;if\;w\cdot x+b>0 \end{array}\right.$$

*w* and *x* are vectors of weights and inputs, respectively. A sigmoid neuron (based on the sigmoid function) was utilized to work with real values and training methods (Equation (3)), providing an output from 0 to 1. This is ideal for representing and estimating a plasticity model due to its data representation flexibility. Furthermore, it allows for gradual learning due to its differentiability and smoothness, adjusting weights and biases gradually (Equation (4)) to minimize error optimally. Additionally, the sigmoid function was harnessed to effectively capture non-linearities owing to its ability to adapt to variations in input data.

(3) $$Output=\sigma \left(w\cdot x+b\right);\;\sigma \left(z\right)=\frac{1}{1+e^{-z}}$$

In Equation (3), because of its smoothness (in the sense that an evolution from −∞ to +∞ for *z* leads to an evolution from 0 to 1 of the output), small changes in the weights generate small changes in the output. To determine the number of hidden layers and neurons, a heuristic approach was implemented, and the cost function of Equation (4) was applied for the training of the artificial neural network.

(4) $$C\left(w,b\right)=\frac{1}{2n_{T}}\sum_{x}{\left\|y\left(x\right)-a(x)\right\|}^{2}$$

*y*(*x*) is the vector with expected output values, *a*(*x*) is the vector of outputs, *x* is the input, and *n_T_* is the total number of inputs for training. The minimization of cost function updates weights and biases at a specific learning rate η according to Equation (5):

(5) $$w^{\prime }=w-\eta \frac{\partial C}{\partial w}\;;\;b^{\prime }=b-\eta \frac{\partial C}{\partial b}$$

To accelerate the learning process, the total average of the gradients is approximated with an average of a smaller number of randomly chosen gradients or mini-batch (Equation (6)):

(6) $$\frac{\sum_{j=1}^{m}\nabla C_{x_{j}}}{m^{\prime }}\;\approx \frac{\sum_{x}\nabla C_{x}}{n}=\nabla C$$

where *n_T_* is the number of training inputs or batch and *m’* is the mini-batch (a number much smaller than *n_T_*). Therefore, Equation (5) becomes:

(7) $$w^{\prime }=w-\frac{\eta }{m^{\prime }}\sum_{j}\frac{\nabla C_{x_{j}}}{\partial w};\;b^{\prime }=b-\frac{\eta }{m^{\prime }}\sum_{j}\frac{\nabla C_{x_{j}}}{\partial b}$$

Each time all the training data is used, a training epoch passes. The next epoch will use the same updated weights and biases in the previous epoch, with the same training data. Since the information is given to the network randomly, weights and biases are readjusted for each epoch. The number of epochs, the value of the learning rate, and the size of the mini-batch must be properly selected, as these factors directly affect the training speed and the quality of the neural network.

### 2.2. Database Generation, Training, and Calibration Strategy Using ANN Configurations

**Figure 2:**
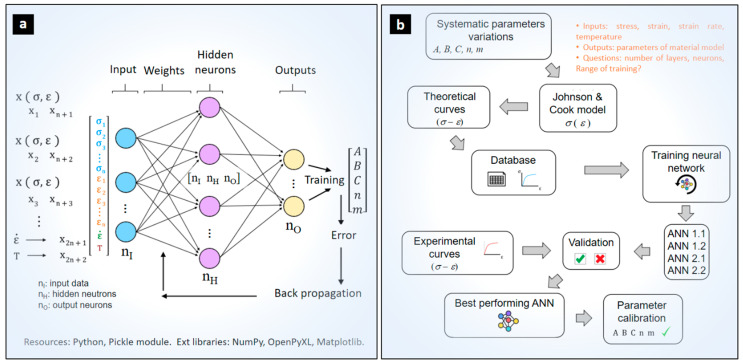
(**a**) Generalized ANN-based calibration strategy of model parameters. (**b**) Training and validation scheme of the studied ANN architectures.

Figure 2a shows the scheme of the implemented neural network architectures for calibrating the material model parameters. First, the input layer data is given as a vector containing the known or experimentally determined flow stresses and strains, for specific strain rate and temperature. Next, hidden layers process the data according to the modeling described in Section 2.1 resulting in a set of material model constants (*A*, *B*, *C*, *n*, *m*). 

The various configurations of artificial neural networks investigated (Table 1) are trained using a database of theoretical stress–strain curves generated by the Johnson–Cook model (Figure 2b). First, training data is generated using a parametric variation of the reference material constants (*A*, *B*, *C*, *n*, and *m*) within a range of approximately ±30%. This ensures that the evaluated Ti64 material remains within a realistic training domain and helps avoid inappropriate prediction behaviors, such as overfitting and underfitting. The selected strain rate ranges and homologous temperature ratios produced 1458 discretized flow curves (Table 1). This database is applied for training the different configurations. In each iteration during training, the weights and biases of the network are updated by applying the gradient descent algorithm. A mini-batch of 10 different randomly chosen input curves is applied until 2000 training epochs are completed. Finally, for each training epoch the neural network will iterate and update its weight and bias values about 486 times. The four ANN configurations are trained with the same learning parameters: 2000 epochs, a mini-batch size of 10, and a learning rate of 3. 

The data processing, design, and training of the neural networks are performed in the open-source programming language Python 3.8.6. The Python libraries used are: NumPy for multidimensional arrays and operation of array data structures [42], OpenPyXL to link data from MS Excel files [43], and Matplotlib for high quality data visualization [44]. 

## 3. Results and Discussion

The global mean absolute percentage error (GMAPE) defined in Equation (8) evaluates the learning quality of the network during training. The number of material model parameters is *n_m_* = [1, …, 5], the number of input curves is *N* = [1, 2, 3], the target parameter for generating the training database is *Xo*, and the parameter computed by the network is *Xc*. Similarly, the learning performance of the network in predicting the flow behavior of the material can be evaluated by substituting the target parameter (*Xo*) for the flow stress generated from the target parameter. At the same time, *Xc* is replaced by the flow stress generated from the ANN-computed material model constants. In this form, *n* is associated with the incremental strain values for each compared flow stress. According to a preliminary result analysis, this GMAPE based on flow stress values is more accurate for evaluating the performance of the ANN, because it directly compares the target flow stress prediction behavior, rather than the model constants affecting the flow stress with different sensitivities. The two approaches are assessed, leading to very different results; for instance, in ANN 1.1, the most straightforward and fastest GMAPE(constants) can lead to 20% errors while GMAPE(stress) reduces its value to 5.53%.

(8) $$GMAPE=\frac{1}{N}\sum_{j=1}^{N}{\left(\frac{1}{n_{m}}\sum_{i=1}^{n_{m}}\frac{\left|{Xo}_{i}-{Xc}_{i}\right|}{{Xo}_{i}}\right)}_{j}$$

### 3.1. Quasi-Static and Dynamic Predictions of Flow Stress with ANN-Based Strategy

**Figure 3:**
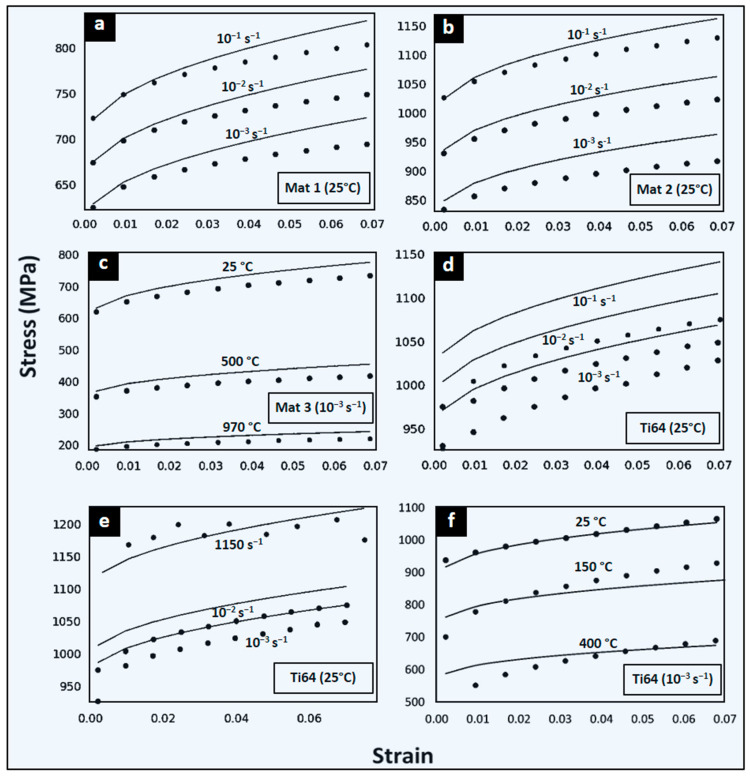
Comparison between input data (dots) and predictions of JC model (lines) calibrated from the ANN-based identification strategy 2.1. Four different materials and six conditions tested. Virtual: (**a**) Mat 1 for [10^−3^, 10^−2^, 10^−1^]/s at 25 °C; (**b**) Mat 2 for [10^−3^, 10^−2^, 10^−1^]/s at 25 °C; and (**c**) Mat 3 at [25, 500, 970] °C at 10^−3^/s. The Ti64 alloy with different input curves are: (**d**) [10^−3^, 10^−2^, 10^−1^]/s at 25 °C; (**e**) [10^−3^, 10^−2^, 1150]/s at 25 °C; and (**f**) [25, 150, 400] °C at 10^−3^/s.

For the dynamic range, the stress–strain curves at 25 °C and for strain rates of 0.001, 0.01, and 10,000/s are compared with the results provided by the material model with the ANN-identified constants (Figure 3). The 66 input neuron configurations of ANN 2.1 and 2.2 are equivalent to 10 points in the input flow stress data. As observed from the global error summary (GMAPE), this configuration provides the best prediction results (Table 2). 

ANN 2.1 and 2.2 present 66 input neurons, corresponding to 10 stress values per data curve. The prediction results for the sets of theoretical and experimental curves of ANN 2.1 are shown in Figure 3. The three investigated virtual materials at different conditions are provided in Figure 3a–c, while Figure 3d–f depicts the real experimental Ti64 material. Table 1 identifies the conditions for each case tested. ANN 2.1 presents a lower prediction error for Mat 1 and Mat 2 than the ANN 1.1 and 1.2 results (Table 2). A better estimation is also observed for ANN 2.1 for dynamic strain rate ranges and wide strain rate ranges, with a slight decrease for quasi-static strain rates. The lowest prediction accuracy for ANN 2.1 is 93.5% (GMAPE of 6.5%) for Mat 3 at low strain rate conditions and large temperature range between 25 and 970 °C (Figure 3c). Note that the input of only three experimental stress–strain curves is required to obtain an adequate set of model parameters. A particular attention is for model predictions of Figure 3e, showing an accuracy of 97.3% (GMAPE of 2.7%) at a wide strain rates range between 10^−3^ and 10^3^ s^−1^.

In general, ANN 2.1 predictions are good and reliable. For Ti64 at ambient temperature, the prediction is accurate and comparable to direct optimization methods of Section 3.2. For the trained ANNs, there is a dependence on the JC model flexibility to adequately represent the observed material behavior. 

### 3.2. Assessment of JC Model in Superplasticity

The ANN has been validated for the large range of strain rates and temperatures below 400 °C. To investigate the applicability of the JC model for training ANN at high temperature ranges, a parametric analysis of the material model is performed. The generalized reduced gradient (GRG) nonlinear optimization for parameters calibration of JC model using minimization of GMAPE is applied. 

**Figure 4:**
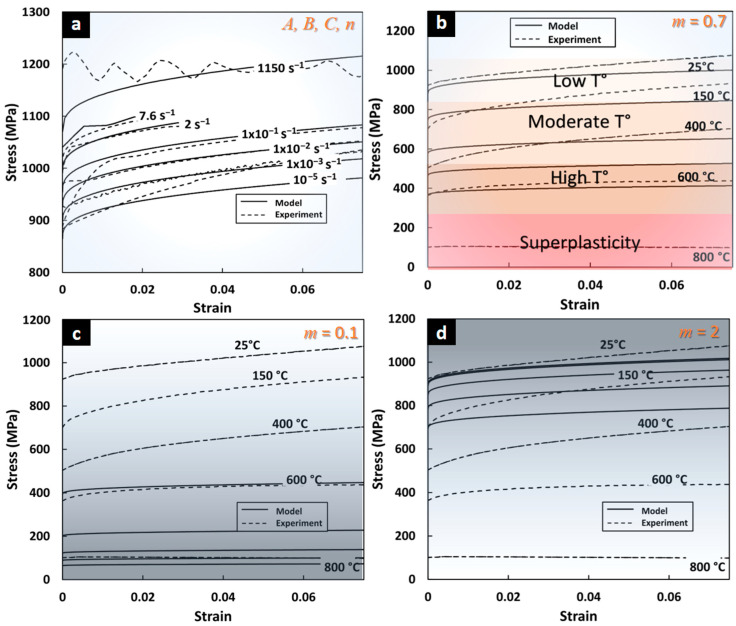
Predictions of Johnson–Cook model from optimization with non-linear GRG. (**a**) Dynamic and static strain flow stress prediction between 10^−5^ s^−1^ and 10^3^ s^−1^ with GMAPE of 1.6%. (**b**) Thermal softening predictions between 25 °C and 800 °C (GMAPE of 4.9% between 25° and 400 °C). Flow predictions for (**c**) low *m* = 0.1 and (**d**) high *m* = 2 show model limitations from (1 − T*^m^) factor.

In terms of strain rate sensitivity at ambient temperature, Figure 4a confirms the capability of the JC model to adequately predict the flow stress in the completely dynamic and static domains. The direct calibration technique (GRG nonlinear optimization) provides a JC model for Ti64 with high accuracy in terms of the strain rate sensitivity for the range of 10^−5^ and 1000 s^−1^. Thermal softening for *T* range between 25 °C and 400 °C also shows good agreement with experimental data (Figure 4b) with a GMAPE of 4.9%. 

Despite high accuracy for low and moderate temperatures, a lack of predictive capability of JC for Ti64 at high temperatures and superplasticity was found. Thermal flow stress driven by the model factor (1 − T*^m^) requires a modifying term for more flexibility. The lowest bound value of softening parameter (*m* → 0 in Figure 4b) integrated in the model factor transform the stress to null, while higher values than the *m* = 2 have almost no effect on the flow stress (upper bound of *m* in Figure 4d). The *m*-value of 0.7 for the range until 400 °C provides the closest JC prediction to experimental flow stress (Figure 4c). The low flexibility of the model factor (1 − T*^m^) in Equation (1) and the restriction of m-value as a constant value for all ranges, is the cause of no solution for an optimal *m*-value of Ti64 in a high temperature range and superplasticity. This JC model constraint does not allow researchers to provide accurate data for ANN training. Other models such as modified Zerilli–Armstrong and Khan–Huang–Liang should be explored in future studies [45]. Finally, the GRG nonlinear optimization method for model parameters calibration reducing GMAPE demonstrates to provide good accuracy in a large range of strain rates: dynamic and static and at low to moderate temperatures. The resulting GMAPE of 5% indicates that the GRG method is comparable to the proposed ANN-based calibration strategy.

## 4. Conclusions

In this work, the development of an ANN-based strategy for calibrating strain rate sensitive material models of equivalent flow stress vs. equivalent strain has been achieved. From the analysis of results, the following statements are drawn:

- The implemented method applied to the parameter calibration of the Johnson–Cook model with the proposed feed-forward ANN architecture of 66 inputs, 30 hidden, and 5 output neurons provides the most accurate results. Input of only three experimental stress–strain curves at different strain rates is required to obtain an adequate set of model parameters with a prediction accuracy of 96.5% (GMAPE of 4.5%).
- The analysis and optimization of the JC model indicates that a high accuracy of the flow stress strain response for the Ti64 alloy is achieved for strain rate range between 10^−3^ and 1000 s^−1^, and for temperatures between 25 and 400 °C, with a prediction accuracy of 95% (GMAPE of 5%).
- The prediction accuracy of an ANN-based calibration strategy of JC flow stress model is slightly better than the direct optimization method, and requires less number of input stress–strain curves.
- Superplastic behavior and moderate temperature (above 400 °C) is not adequately modeled by JC. This finding is also similar to the calibration results using other models such as Northon–Hoff [40].

Further work should be focused on the following three lines: (a) investigating the flexibility of the ANN strategy using virtual data generated from several material models and simulations for more robust training; (b) exploring a hybrid ANN-constitutive model for predicting plastic behavior in multiaxial stress and multiaxial strain states; and (c) developing an ANN-based material model trained with complex experimental data and implementing the ANN-derived expressions as a smart element in finite element codes [46,47].

## Figures and Tables

**Table 1:** Configuration of the investigated ANNs and features of discretized flow stress–strain data generation for the training stage.

ANN	Number of Input σ-ε Curves	ε Range [mm/mm]	Discrete Points per Curve	Discrete Strain Step	Number of Hidden Layers	Neurons per Layer
1.1	3	[0.002–0.075]	74	0.001	1	[450 30 5]
1.2					2	[450 30 15 5]
2.1			10	0.0074	1	[66 30 5]
2.2					2	[66 30 15 5]

**Table 2:** Global accuracy of ANN-based calibration configurations for 3 virtual materials and the Ti64 alloy tested at different conditions.

	Input Material Dataset	Input Stress–Strain Data		GMAPE by ANN Configuration (%)			
Material	[A, B, C, n, m]	Strain Rate (s^−1^)	T (°C)	ANN 1.1 [450 30 5]	ANN 1.2 [450 30 15 5]	ANN 2.1 [66 30 5]	ANN 2.2 [66 30 15 5]
Mat 1	[630, 252, 0.0105, 0.28, -]	[10^−3^, 10^−2^ 10^−1^]	[25, 25, 25]	5.5	9.7	2.0	4.1
Mat 2	[900, 360, 0.015, 0.52, -]	[10^−3^, 10^−2^, 10^−1^]	[25, 25, 25]	3.0	7.5	2.7	6.2
Mat 3	[630, 468, 0.0105, 0.4, 0.7]	[10^−3^, 10^−3^, 10^−3^]	[25, 500, 970]	7.4	8.3	6.5	9.1
Ti64	(Exp. data)	[10^−3^, 10^−2^, 10^−1^]	[25, 25, 25]	19	23	5.2	2.7
		[10^−3^, 10^−2^, 1150]	[25, 25, 25]	14	14	2.6	2.5
		[10^−3^, 10^−3^, 10^−3^]	[25, 150, 400]	13	9.2	3.6	3.9

## Data Availability

Supporting information is available from the author upon reasonable request.
